# Structures of phosphopantetheine adenylyltransferase from *Burkholderia pseudomallei*

**DOI:** 10.1107/S1744309111004349

**Published:** 2011-08-13

**Authors:** Thomas E. Edwards, David J. Leibly, Janhavi Bhandari, Jacob B. Statnekov, Isabelle Phan, Shellie H. Dieterich, Jan Abendroth, Bart L. Staker, Wesley C. Van Voorhis, Peter J. Myler, Lance J. Stewart

**Affiliations:** a Seattle Structural Genomics Center for Infectious Disease (SSGCID), USA; b Emerald BioStructures, 7869 NE Day Road West, Bainbridge Island, WA 98110, USA; c Department of Medicine, Division of Allergy and Infectious Diseases, MS 356423, School of Medicine, University of Washington, Seattle, WA 98195-6423, USA; d Seattle Biomedical Research Institute, 307 Westlake Avenue North, Suite 500, Seattle, WA 98109, USA; e Departments of Global Health and Medical Education and Biomedical Informatics, University of Washington, Seattle, WA 98195, USA

**Keywords:** biosynthesis, CoaD, coenzyme A, *Burkholderia*, infectious diseases, melioidosis, nucleotidyltransferases, pantetheine-phosphate adenylyltransferase, phosphopanthetheine adenylyltransferase, PPAT, Rossman fold

## Abstract

Phosphopantetheine adenylyltransferase (PPAT) catalyzes the fourth of five steps in the coenzyme A biosynthetic pathway, reversibly transferring an adenylyl group from ATP onto 4′-phosphopantetheine to yield dephospho-coenzyme A and pyrophosphate. *Burkholderia pseudomallei* is a soil- and water-borne pathogenic bacterium and the etiologic agent of melioidosis, a potentially fatal systemic disease present in southeast Asia. Two crystal structures are presented of the PPAT from *B. pseudomallei* with the expectation that, because of the importance of the enzyme in coenzyme A biosynthesis, they will aid in the search for defenses against this pathogen. A crystal grown in ammonium sulfate yielded a 2.1 Å resolution structure that contained dephospho-coenzyme A with partial occupancy. The overall structure and ligand-binding interactions are quite similar to other bacterial PPAT crystal structures. A crystal grown at low pH in the presence of coenzyme A yielded a 1.6 Å resolution structure in the same crystal form. However, the experimental electron density was not reflective of fully ordered coenzyme A, but rather was only reflective of an ordered 4′-diphosphopantetheine moiety.

## Abstract 2

Phosphopantetheine adenylyltransferase (PPAT) reversibly converts ATP and 4′-phosphopantetheine into dephospho-coenzyme A and pyrophosphate. Crystal structures are presented of PPAT from *B. pseudomallei*, the pathogenic bacterium that causes melioidosis.

## 1. Introduction

Coenzyme A is biosynthesized in five invariant steps from pantothenate (vitamin B_5_), cysteine and ATP (Robishaw & Neely, 1985 ▶). The fourth step in the biosynthetic pathway is the adenylylation of 4′-­phosphopantetheine using ATP to form dephospho-coenzyme A and pyrophosphate (Martin & Drueckhammer, 1993 ▶). This reversible reaction is catalyzed by phosphopantetheine adenylyltransferase, which is also named pantetheine-phosphate adenylyltransferase or PPAT. Dephospho-coenzyme A synthesis by PPAT is believed to be the rate-limiting step in CoA biosynthesis. Because of the importance of coenzyme A in the citric acid cycle as well as fatty-acid synthesis, coenzyme A biosynthetic proteins are believed to be potential drug targets for infectious disease organisms and PPAT inhibitors have been developed (Zhao *et al.*, 2003 ▶).

The first PPAT crystal structures were solved from *Escherichia coli* (PDB entries 1qjc, 1gn8, 1h1t and 1b6t; Izard, 2002 ▶, 2003 ▶; Izard & Geerlof, 1999 ▶; Izard *et al.*, 1999 ▶) and were followed by a number of other PPAT crystal structures from *Archaeoglobus fulgidus* (PDB entry 3do8; R. Zhang, R. Wu, R. Jedrzejczak & A. Joachimiak, unpublished work), *Bacillus subtilis* (PDB entry 1o6b; Badger *et al.*, 2005 ▶), *Mycobacterium tuberculosis* (PDB entry 1tfu; Morris & Izard, 2004 ▶), *Thermotoga maritima* (PDB entry 1vlh; Joint Center for Structural Genomics, unpublished work), *Thermus thermophilus* (PDB entry 1od6; Takahashi *et al.*, 2004 ▶), *Staphylococcus aureus* (PDB entry 3f3m; Lee *et al.*, 2009 ▶) and *Yersinia pestis* (PDB entries 3l92 and 3l93; J. Osipiuk, N. Maltseva, M. Makowska-grzyska, K. Kwon, W. F. Anderson & A. Joachimiak, unpublished work). On the whole, these structures include apo enzymes (PDB entries 3l93, 3do8 and 1tfu), a variety of substrate-bound states (*e.g.* 4′-phosphopantetheine in PDB entries 1od6 and 1qjc and ATP in PDB entry 1gn8) and product states (dephospho-coenzyme A), as well as non-native states (*e.g.* ADP, coenzyme A).

*Burkholderia pseudomallei* is a pathogenic bacterium that causes the potentially fatal disease melioidosis (Cheng, 2010 ▶). *B. pseudomallei* is closely related to *B. mallei*, the organism that causes glanders, and more distantly related to *B. cenocepacia*, an organism that causes acute infections in patients with cystic fibrosis. In *B. pseudomallei*, the *coaD* gene encodes the 166-residue protein *Bp* PPAT, although it has not yet been shown that *Bp* PPAT is essential for *B. pseudomallei*. Here, we present two crystal structures of *Bp* PPAT. One structure appears to contain dephospho-coenzyme A from the expression host carried through the protein purification. A second structure grown in the presence of coenzyme A only showed significant electron density for the 4′-diphosphopantetheine moiety and weaker electron density for the adenine nucleobase.

## 2. Methods

### 2.1. Protein expression and purification

Phosphopantetheine adenylyltransferase from *B. pseudomallei* strain 1710b (NCBI YP 332162.1; *coaD* gene BURPS1710B_0748; UniProt Q3JW91; Pfam ID PF01467; EC 2.7.7.3) spanning the full-length protein from residues 1–166 (‘ORF’) was cloned into a pAVA0421 vector encoding an N-terminal histidine-affinity tag followed by the human rhinovirus 3C protease-cleavage sequence (the entire tag sequence is MAHHHHHHMGTLEAQTQGPGS-ORF) using ligation-independent cloning (Aslanidis & de Jong, 1990 ▶; Kelley *et al.*, 2011 ▶). Phosphopantetheine adenylyltransferase was expressed in *E. coli* using BL21(DE3)R3 Rosetta cells and autoinduction medium (Studier, 2005 ▶) in a LEX Bioreactor (Leibly *et al.*, 2011 ▶). The frozen cells were resuspended in lysis buffer (25 m*M* HEPES pH 7.0, 500 m*M* NaCl, 5% glycerol, 30 m*M* imidazole, 0.025% azide, 0.5% CHAPS, 10 m*M* MgCl_2_, 1 m*M* TCEP, 250 ng ml^−1^ protease inhibitor AEBSF and 0.05 µg ml^−1^ lysozyme) at 277 K. The resuspended cell pellet was disrupted on ice for 30 min with a Virtis sonicator (Virtis 408912; settings: 100 W power with alternating cycles of 15 s pulse-on and 15 s pulse-off). The cell debris was incubated with 20 µl Benzonase nuclease (25 units µl^−1^) at room temperature for 45 min and clarified by centrifugation on a Sorvall SLA-1500 at 14 000 rev min^−1^ for 75 min at 277 K. The protein was purified from the clarified cell lysate by immobilized metal-affinity chromatography (IMAC) on a His Trap FF 5 ml column (GE Healthcare) equilibrated with binding buffer (25 m*M* HEPES pH 7.0, 0.5 *M* NaCl, 5% glycerol, 30 m*M* imidazole, 0.025% azide, 1 m*M* TCEP) at 277 K. The recombinant protein was eluted with binding buffer supplemented with 250 m*M* imidazole. The affinity tag was removed by incubation with His_6_-MBP-3C protease at 277 K during dialysis into binding buffer overnight, followed by a subtractive nickel gravity-flow column with the buffers described above. The now tagless protein (sequence GPGS-ORF) was collected in the flowthrough and was further resolved by size-exclusion chromatography (SEC) using a HiLoad 26/60 Superdex 200 column (GE Healthcare) at 277 K. Pure fractions collected in SEC buffer (25 m*M* HEPES pH 7.0, 0.5 *M* NaCl, 2 m*M* DTT, 0.025% azide and 5% glycerol) as a single peak were pooled. During concentration at 277 K, the protein was observed to precipitate. 10 m*M* ATP (Sigma–Aldrich, >99% purity) was added to the protein solution, which allowed concentration of the protein to 5.5 mg ml^−1^. The protein sample was flash-frozen and stored at 193 K. A second batch of protein was prepared in which the affinity tag was not removed. This purification used more optimal buffers identified by thermal denaturation studies. To improve the buffer conditions for the second purification, nickel IMAC-purified protein from the first batch was subjected to an 80-buffer thermal denaturation screen. 12.5 µg protein was added to 26 µl buffer mixed with SYPRO Orange protein dye (Invitrogen). Thermal denaturation was performed over a gradient from 293 to 373 K as described by Crowther *et al.* (2010 ▶). The buffer conditions that showed the largest positive shift in thermal denaturation temperature were selected for use in purification of the second batch of protein. Cells were lysed as in the first purification batch except in an optimized lysis buffer (10 m*M* Tris pH 8.0, 0.5 *M* NaCl, 5% glycerol, 30 m*M* imidazole, 0.025% azide, 0.5% CHAPS, 10 m*M* MgCl_2_, 250 ng ml^−1^ AEBSF and 0.05 µg ml^−1^ lysozyme). IMAC was performed as in the first preparation except with optimized binding buffer (10 m*M* Tris pH 8.0, 0.5 *M* NaCl, 5% glycerol, 30 m*M* imidazole, 1 m*M* MgCl_2_). The protein was eluted with optimized binding buffer supplemented with 250 m*M* imidazole. SEC was performed with an optimized SEC buffer (10 m*M* Tris pH 8.0, 450 m*M* NaCl, 5% glycerol, 1 m*M* MgCl_2_). Fractions containing the protein were pooled, coenzyme A (Sigma-Aldrich catalog No. C3144, >85% purity) was added to 1 m*M* and the protein was concentrated to 19.2 mg ml^−1^ with good solubility. The protein sample was flash-frozen and stored at 193 K prior to crystallography. Each protein sample was >95% pure as determined by denaturing SDS–PAGE.

### 2.2. Crystallization

Sitting-drop vapour-diffusion crystallization trials were set up at 289 K using either the JCSG+, PACT (Newman *et al.*, 2005 ▶) or Cryo Full sparse-matrix screens from Emerald BioSystems or the Crystal Screen HT sparse-matrix screen from Hampton Research. *Bp* PPAT stock solutions (0.4 µl) were mixed with reservoir solution (0.4 µl) and equilibrated against reservoir solution (100 µl) using 96-well Compact Jr plates from Emerald BioSystems. Crystals grew in several conditions, but those used in X-ray data-collection and structure determination were obtained from Crystal Screen HT conditions C8 (protein sequence GPGS-ORF, 5.5 mg ml^−1^ protein solution equilibrated against 2.0 *M* ammonium sulfate, which resulted in PDB entry 3pxu) and F1 (protein sequence MAHHHHHHMGTLEAQTQG­PGS-ORF, 19.2 mg ml^−1^ protein solution equilibrated against 0.2 *M* ammonium sulfate, 0.1 *M* sodium acetate pH 4.6, 30% PEG 2000 MME, which resulted in PDB entry 3k9w).

### 2.3. Data collection and structure determination

The crystals grown in the presence of 2.0 *M* ammonium sulfate were harvested after cryoprotection in lithium sulfate. A data set was collected in-house using a Rigaku SuperBright FR-E+ rotating-anode X-ray generator with Osmic VariMax HF optics and a Saturn 944+ CCD detector (Table 1 ▶). The data were reduced with *HKL*-2000 (Minor *et al.*, 2006 ▶). The structure was solved by molecular replacement using *Phaser* (McCoy *et al.*, 2007 ▶) from the *CCP*4 suite (Winn *et al.*, 2011 ▶) using the protein model of molecule *A* of the *E. coli* PPAT crystal structure (PDB entry 1b6t; Izard & Geerlof, 1999 ▶) as the search model. The structure was initially rebuilt with *ARP*/*wARP* (Langer *et al.*, 2008 ▶), followed by numerous reiterative rounds of refinement in *REFMAC* (Murshudov *et al.*, 1997 ▶) and manual building in *Coot* (Emsley & Cowtan, 2004 ▶). The final model contained one copy of *Bp* PPAT spanning residues Ser0 (from the expression-tag remnant) through Ala91 and residues Phe95 through Ala161, two sulfate ions, two glycerol molecules, 90 water molecules and dephospho-coenzyme A. The low-pH crystals were harvested after cryoprotection with 20% ethylene glycol and 80% precipitant. A data set was collected on the Canadian Light Source beamline 08ID-1 (Table 1 ▶). The data were reduced with *HKL*-2000 (Minor *et al.*, 2006 ▶) and the structure was solved by refinement against the protein model of the first structure. The final model was produced after numerous reiterative rounds of refinement in *REFMAC* (Murshudov *et al.*, 1997 ▶) and manual building in *Coot* (Emsley & Cowtan, 2004 ▶). The final model contained one copy of *Bp* PPAT spanning residues Ser0 through Ala161, a sulfate ion, an acetate ion, a polyethylene glycol molecule, 137 water molecules, adenine and 4′-diphosphopantetheine. For both structures water molecules were built with stringent criteria of electron density above 1.2σ in the 2|*F*_o_| − |*F*_c_| map and one or more hydrogen-bonding partners. Although the *R*_merge_ values for both structures (Table 1 ▶) may be high by some standards, inclusion of data to higher resolution improved the experimental electron-density maps for both structures and allowed improved model building relative to more conservative resolution limits. The final model for each structure showed good geometry and fitness (Table 2 ▶) according to analysis with *MolProbity* (Chen *et al.*, 2010 ▶).

## 3. Results and discussion

### 3.1. Overall structure

**Figure 1:**
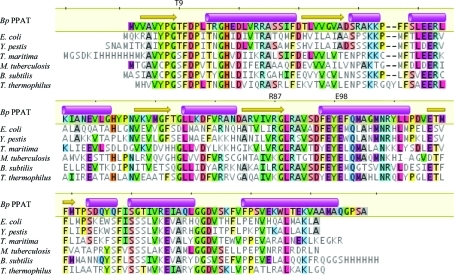
Multiple sequence alignment of bacterial PPATs. Sequences are shown from *B. pseudomallei* (*Bp* PPAT; PDB entry 3pxu; present study), *E. coli* (PDB entry 1b6t; Izard & Geerlof, 1999 ▶), *Y. pestis* (PDB entry 3l93; Osipiuk *et al.*, unpublished work), *T. maritima* (PDB entry 1vlh; Joint Center for Structural Genomics, unpublished work), *M. tuberculosis* (PDB entry 1tfu; Morris & Izard, 2004 ▶), *B. subtilis* (PDB entry 1o6b; Badger *et al.*, 2005 ▶) and *T. thermophilus* (PDB entry 1od6; Takahashi *et al.*, 2004 ▶). α-­Helices and β-sheets from the *Bp* PPAT structure are shown as magenta cylinders and yellow arrows, respectively. The side chains of Thr9, Arg87 and Glu98 interact with dephospho-coenzyme A in the 3pxu structure. This figure was prepared with *Geneious* (Drummond *et al.*, 2010 ▶).

**Figure 2:**
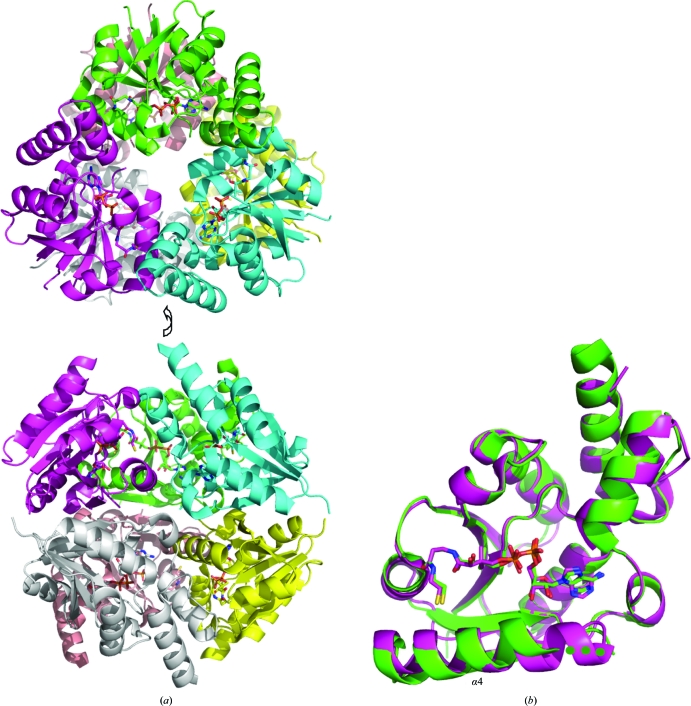
Crystal structure of *Bp* PPAT solved at 2.1 Å resolution. (*a*) Biologically relevant hexameric structure of *Bp* PPAT from a crystal structure with bound dephospho-coenzyme A solved at 2.1 Å resolution. (*b*) Overlay of the dephospho-coenzyme A-bound structures of *Bp* PPAT (green) and *E. coli* PPAT (PDB entry 1b6t, magenta; Izard & Geerlof, 1999 ▶). Green spheres are used to illustrate disordered residues in the *Bp* PPAT crystal structures. Figs. 2 and 3 were prepared using *PyMOL* (DeLano, 2002 ▶).

*Bp* PPAT has approximately 42–50% sequence identity (64–75% similarity) to PPATs from *B. subtilis*, *E. coli*, *M. tuberculosis*, *T. maritima*, *T. thermophilus* and *S. aureus* (Fig. 1 ▶). In contrast, *Bp* PPAT has significantly lower sequence identity to other PPATs such as those from *A. fulgidus* and *Y. pestis*. The crystal structure of *Bp* PPAT features a Rossmann fold, which is known to bind di­nucleotides as well as GTP and ATP. One copy of *Bp* PPAT was observed in the asymmetric unit, indicating that the other five copies that comprise the biologically active homohexamer are crystallo­graphically equivalent. The homohexameric quaternary structure of *Bp* PPAT (Fig. 2 ▶ *a*) is similar to other members of the nucleotidyltransferase superfamily and all other reported PPAT crystal structures.

### 3.2. Product state

From a protein sample concentrated in the presence of ATP, we solved a 2.1 Å resolution crystal structure of *Bp* PPAT (Table 1 ▶). This structure had clear evidence but weak electron density for de­phospho-coenzyme A in the active site, indicating that dephospho-coenzyme A was only partially occupied. Coenzyme A metabolites are known to exist at significant concentrations in *E. coli* (Jackowski & Rock, 1984 ▶) and thus it is not surprising to see dephospho-coenzyme A carried through the purification from the expression host. Refinement with the occupancy of dephospho-coenzyme A set to 0.5 (*i.e.* 50%) resulted in crystallographic *B* factors that were in line with those of the surrounding protein atoms (∼30 Å^2^). In the *E. coli* PPAT crystal structure dephospho-coenzyme A was present in only one trimer, while the other trimer was unliganded (Izard & Geerlof, 1999 ▶). Overall, the *Bp* PPAT and *E. coli* PPAT dephospho-coenzyme A-bound crystal structures are quite similar (C^α^ r.m.s.d. of 1.00 Å; Fig. 2 ▶ *b*). Dephospho-coenzyme A forms many packing interactions and hydrogen bonds with backbone amides in the active site, but also makes hydrogen bonds to the side chains of the conserved residues Thr9 (which is conserved as a threonine or serine), Arg87 (which is conserved as an arginine or lysine) and Glu98 (see Fig. 1 ▶ for sequence conservation). Differences were observed between the *E. coli* PPAT unliganded and coenzyme A-bound protomers, especially the movement of residues at the N-terminal side of α4. Equivalent residues in the *Bp* PPAT structure (92–94) are disordered in the product state and several neighboring residues have disordered side chains (Phe95, Phe99 and Tyr107; Fig. 2 ▶ *b*). It is unknown whether *Bp* PPAT follows asymmetric ligation in the same manner as *E. coli* PPAT (Izard & Geerlof, 1999 ▶) and *M. tuberculosis* PPAT (Morris & Izard, 2004 ▶).

### 3.3. Structure at low pH

**Figure 3:**
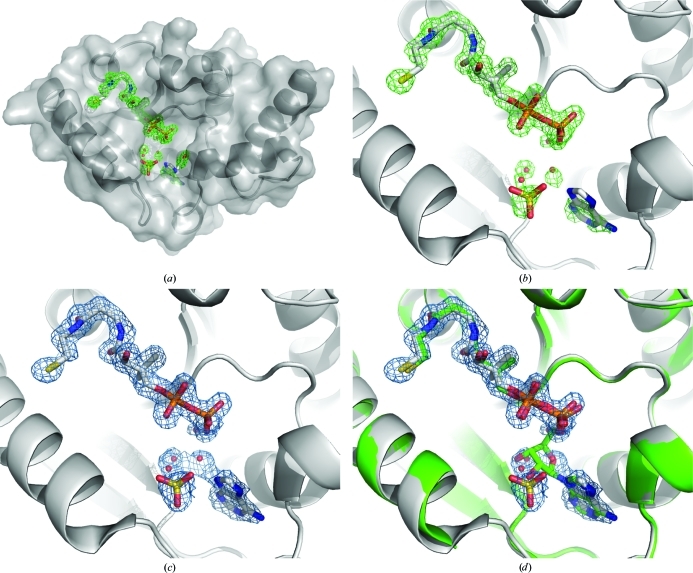
Crystal structure of *Bp* PPAT solved at 1.6 Å resolution. (*a*) Monomeric structure of *Bp* PPAT in ribbon format with molecular-surface rendering in light transparency. Active-site components are modelled in stick representation and selected waters are shown as spheres. The active-site ligand-omit density map (|*F*_o_| − |*F*_c_|) is shown as a green mesh contoured at 3.0σ. (*b*) Close-up of the active site. The final protein model is shown in ribbon representation along with 4′-diphosphopantetheine, adenine and a sulfate ion in stick representation and selected waters as red spheres. The active-site ligand-omit electron-density map (|*F*_o_| − |*F*_c_|) is shown as a green mesh contoured at 3.0σ. (*c*) Active site as in (*b*) with the 2|*F*_o_| − |*F*_c_| electron-density map shown as a blue mesh contoured at 1.0σ. (*d*) Overlay of the final refined structure of *Bp* PPAT from (*c*) along with the structure of *Bp* PPAT-bound dephospho-coenzyme A solved at 2.1 Å resolution with coloring as in Fig. 2 ▶(*b*).

We prepared a second *Bp* PPAT protein sample that contained the full-length expression tag and which was concentrated in the presence of coenzyme A (see §[Sec sec2.1]2.1). From this sample, we obtained a crystal at low pH (4.6) that resulted in a 1.6 Å resolution data set of the same crystal form as the dephospho-coenzyme-A-bound structure (Table 1 ▶). Since this structure obtained from a protein sample containing the N-terminal His tag and the product-state structure described above resulted in isomorphous crystal forms, it appears that the presence of the N-terminal His tag does not affect the structure of *Bp* PPAT. This data set had omit electron density reminiscent of coenzyme A, with strong density for the pantetheine and diphosphate moieties, but had little or no omit electron density for what should have been the adenine ring, ribose ring and 3′-phosphate (Figs. 3*a* ▶ and 3 ▶ *b*). Refinement with coenzyme A in the active site resulted in poor geometry of coenzyme A bonds, with a strong negative peak in the |*F*_o_| − |*F*_c_| map centered on C4–C5 of the ribose ring. This strong negative peak led us to believe that coenzyme A had been hydrolyzed or was disordered beyond the diphosphate moiety. Refinement with 4′-diphosphopantetheine gave a significantly better fit with excellent electron density (Fig. 3 ▶ *c*) and crystallographic *B* factors that were on a par with the surrounding protein atoms (Table 2 ▶). We noted modest omit electron density for the adenine ring, which was also modelled (Fig. 3 ▶ *c*) and resulted in reasonable electron density and somewhat higher average *B* factors than the surrounding protein atoms, implying that the adenine ring may only be partially occupied. The remaining electron-density features fit well as three waters and a sulfate ion (the crystal grew in the presence of 0.2 *M* sulfate ion). Moreover, the adenine and 4′-diphosphopantetheine components overlay well with the *Bp* PPAT structure solved in the presence of dephospho-coenzyme A (Fig. 3 ▶ *d*). We note that the feature modelled as a sulfate ion is unlikely to be the 3′-phosphate of coenzyme A, since it appears off the 2′ position when overlaid with the structure containing dephospho-coenzyme A.

## 4. Conclusions

We obtained high-resolution crystal structures of phosphopantetheine adenylyltransferase from *B. pseudomallei* with the reaction product dephospho-coenzyme A and from a second crystal obtained in the presence of coenzyme A. The structure obtained of the product state is similar to other bacterial PPAT crystal structures obtained in the product state. The crystal grown at low pH in the presence of coenzyme A resulted in a structure solution that showed clear electron density for the 4′-diphosphopantetheine and adenine moieties. It is unknown whether the lack of electron density for the ribose ring and 3′-phosphate resulted from hydrolysis under the crystallographic conditions, hydrolysis by the enzyme or is reflective of disorder of the coenzyme.

## Supplementary Material

PDB reference: PPAT, 3pxu

PDB reference: 3k9w

## Figures and Tables

**Table 1:** Data-collection statistics Values in parentheses indicate the values for the highest of 20 resolution shells.

Ligand	Dephospho-CoA	Partial CoA
Space group	*I*432	*I*432
Unit-cell parameters (Å)	*a* = *b* = *c* = 134.2	*a* = *b* = *c* = 134.4
Wavelength (Å)	1.5418	0.97953
Resolution range (Å)	50–2.1 (2.18–2.10)	50–1.6 (1.66–1.60)
Unique reflections	12286	27383
Multiplicity	28.3 (22.4)	10.7 (9.1)
Completeness (%)	99.7 (98.1)	99.0 (100)
*R*_merge_†	0.084 (0.519)	0.067 (0.555)
Mean *I*/σ(*I*)	44.5 (6.6)	32.8 (4.0)

† *R*_merge_ = 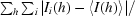 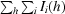.

**Table 2:** Refinement and model statistics Values in parentheses indicate the values for the highest of 20 resolution shells.

Ligand	Dephospho-CoA	Partial CoA
Resolution range (Å)	50–2.1 (2.18–2.10)	50–1.6 (1.66–1.60)
*R*_cryst_	0.202 (0.204)	0.192 (0.229)
*R*_free_†	0.242 (0.315)	0.207 (0.266)
R.m.s.d. bonds (Å)	0.015	0.008
R.m.s.d. angles (°)	1.463	1.193
Protein atoms	1254	1360
Nonprotein atoms	155	192
Mean *B* factor (Å^2^)	30.0	15.1
Ligand *B* factor (Å^2^)	33.2	14.5
Residues in favored region (%)	98.7	99.4
Residues in allowed region (%)	100	100
*MolProbity* score [percentile]	1.36 [99th]	1.47 [90th]
PDB code	3pxu	3k9w

† *R*_free_ = 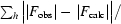 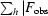. The free *R* factor was calculated using 5% of the reflections omitted from the refinement (Winn *et al.*, 2011 ▶).

## References

[bb1] Aslanidis, C. & de Jong, P. J. (1990). *Nucleic Acids Res.* **18**, 6069–6074. 10.1093/nar/18.20.6069 PMC332407 2235490

[bb2] Badger, J. *et al.* (2005). *Proteins*, **60**, 787–796. 10.1002/prot.20541 16021622

[bb3] Chen, V. B., Arendall, W. B., Headd, J. J., Keedy, D. A., Immormino, R. M., Kapral, G. J., Murray, L. W., Richardson, J. S. & Richardson, D. C. (2010). *Acta Cryst.* D**66**, 12–21. 10.1107/S0907444909042073 PMC2803126 20057044

[bb4] Cheng, A. C. (2010). *Curr. Opin. Infect. Dis.* **23**, 554–559. 10.1097/QCO.0b013e32833fb88c 20847695

[bb6] Crowther, G. J., He, P., Rodenbough, P. P., Thomas, A. P., Kovzun, K. V., Leibly, D. J., Bhandari, J., Castaneda, L. J., Hol, W. G., Gelb, M. H., Napuli, A. J. & Van Voorhis, W. C. (2010). *Anal. Biochem.* **399**, 268–275. 10.1016/j.ab.2009.12.018 PMC3270315 20018159

[bb7] DeLano, W. L. (2002). *PyMOL* http://www.pymol.org.

[bb8] Drummond, A. J., Ashton, B., Buxton, S., Cheung, M., Cooper, A., Heled, J., Kearse, M., Moir, R., Stones-Havas, S., Sturrock, S., Thierer, T. & Wilson, A. (2010). *Geneious* v.5.1. http://www.geneious.com. 10.1093/bioinformatics/bts199 PMC3371832 22543367

[bb9] Emsley, P. & Cowtan, K. (2004). *Acta Cryst.* D**60**, 2126–2132. 10.1107/S0907444904019158 15572765

[bb10] Izard, T. (2002). *J. Mol. Biol.* **315**, 487–495. 10.1006/jmbi.2001.5272 11812124

[bb11] Izard, T. (2003). *J. Bacteriol.* **185**, 4074–4080. 10.1128/JB.185.14.4074-4080.2003 PMC164871 12837781

[bb12] Izard, T. & Geerlof, A. (1999). *EMBO J.* **18**, 2021–2030. 10.1093/emboj/18.8.2021 PMC1171286 10205156

[bb13] Izard, T., Geerlof, A., Lewendon, A. & Barker, J. J. (1999). *Acta Cryst.* D**55**, 1226–1228. 10.1107/s0907444999004394 10329792

[bb14] Jackowski, S. & Rock, C. O. (1984). *J. Bacteriol.* **158**, 115–120. 10.1128/jb.158.1.115-120.1984 PMC215387 6370952

[bb15] Kelley, A., Hewitt, S. N., Choi, R., Napuli, A. J. & Van Voorhis, W. C. (2011). In preparation.

[bb16] Langer, G., Cohen, S. X., Lamzin, V. S. & Perrakis, A. (2008). *Nature Protoc.* **3**, 1171–1179. 10.1038/nprot.2008.91 PMC2582149 18600222

[bb17] Lee, H. H., Yoon, H.-J., Kang, J. Y., Park, J. H., Kim, D. J., Choi, K.-H., Lee, S.-K., Song, J., Kim, H.-J. & Suh, S. W. (2009). *Acta Cryst.* F**65**, 987–991. 10.1107/S1744309109036616 PMC2765882 19851003

[bb18] Leibly, D. J., Nguyen, T. N., Kao, L. T., Newling, P. A., Le, K. P., Napuli, A. J. & Van Voorhis, W. C. (2011). In preparation.

[bb19] Martin, D. P. & Drueckhammer, D. G. (1993). *Biochem. Biophys. Res. Commun.* **192**, 1155–1161. 10.1006/bbrc.1993.1537 8389542

[bb20] McCoy, A. J., Grosse-Kunstleve, R. W., Adams, P. D., Winn, M. D., Storoni, L. C. & Read, R. J. (2007). *J. Appl. Cryst.* **40**, 658–674. 10.1107/S0021889807021206 PMC2483472 19461840

[bb21] Minor, W., Cymborowski, M., Otwinowski, Z. & Chruszcz, M. (2006). *Acta Cryst.* D**62**, 859–866. 10.1107/S0907444906019949 16855301

[bb22] Morris, V. K. & Izard, T. (2004). *Protein Sci.* **13**, 2547–2552. 10.1110/ps.04816904 PMC2280004 15322293

[bb23] Murshudov, G. N., Vagin, A. A. & Dodson, E. J. (1997). *Acta Cryst.* D**53**, 240–255. 10.1107/S0907444996012255 15299926

[bb24] Newman, J., Egan, D., Walter, T. S., Meged, R., Berry, I., Ben Jelloul, M., Sussman, J. L., Stuart, D. I. & Perrakis, A. (2005). *Acta Cryst.* D**61**, 1426–1431. 10.1107/S0907444905024984 16204897

[bb25] Robishaw, J. D. & Neely, J. R. (1985). *Am. J. Physiol.* **248**, E1–E9. 10.1152/ajpendo.1985.248.1.E1 2981478

[bb26] Studier, F. W. (2005). *Protein Expr. Purif.* **41**, 207–234. 10.1016/j.pep.2005.01.016 15915565

[bb27] Takahashi, H., Inagaki, E., Fujimoto, Y., Kuroishi, C., Nodake, Y., Nakamura, Y., Arisaka, F., Yutani, K., Kuramitsu, S., Yokoyama, S., Yamamoto, M., Miyano, M. & Tahirov, T. H. (2004). *Acta Cryst.* D**60**, 97–104. 10.1107/s0907444903025319 14684898

[bb5] Winn, M. D. *et al.* (2011). *Acta Cryst.* D**67**, 235–242.

[bb28] Zhao, L., Allanson, N. M., Thomson, S. P., Maclean, J. K., Barker, J. J., Primrose, W. U., Tyler, P. D. & Lewendon, A. (2003). *Eur. J. Med. Chem.* **38**, 345–349. 10.1016/s0223-5234(03)00047-3 12750020

